# Nasal Stenosis

**Published:** 1886-01

**Authors:** 


### Nasal Stenosis.

At the meeting of the Mississippi Valley Medical Society, at Evansville, Ind., in September, Dr. G. V. Wollen, of Indianapolis, read a paper on the above subject, the main points of which are given below:

He first called attention to the want of attention in former times to nasal disease, and the very great advance made towards a correct knowledge of it in the last ten years. He then briefly detailed the peculiarities of the anatomical construction of the nasal and accessory cavities, especially adapting them to their physiological functions, viz., olfaction, respiration, conduction of the secretion to the pharynx and as an accessory to articulate speech. The title of the paper called attention only to a symptom, but it and other symptoms often demanded attention as a disease.

Chief causes are hypertrophy of turbinated bodies and mucous membranes, deviations of septum, adenoids, polypi, exastoses and hypertrophied pharyngeal tonsils. The degree of stenosis did not always denote the degree of disturbance, as is now well appreciated in the causation of hay fever.

The disturbance which followed were discussed under the following heads:

1st. Interference with the respiratory act.

2d. With the natural flow of secretion.

3d. With speech and song.

4th. With olfaction.

Interference with nasal breathing soon establishes disease of the parts of the respiratory organ by producing mouth-breathing, a most dangerous practice.

The law of catarrhal diseases is that of descent, so that much that is supposed to originate in the lungs possibly does not.

Our schools are full of children vainly trying to articulate successfully, owing to enlarged nasal and pharyngeal tonsils. The nose is an important resonance chamber, and if by any cause great modification of the tone must ensue, and much that is supposed to be the result of faulty education and physical peculiarities, is, in fact, the result of obstruction of this function.

Other features cf nasal stenosis, such as pressure, irritation, cerebral pains, etc., were noticed, but not discussed for want of time.

Dr. A. C. Bernays, of St. Louis, queried if the essayist had ever seen congenital stenosis of both nose holes.

Dr. Wm. Chatham, of Louisville, thought the essayist had failed to refer, in his paper, to nasal stenosis as a cause of asthma and hay fever. There must be three factors present to produce hay fever: an external irritant, a predisposition on the part of the patient, and a vulnerable or sensitive area, through which the system becomes influenced by the irritant. The "sensitive areas" are located in the region of the distribution of the spheno-palatine ganglion; that is, in the inferior and middle turbinated bones and the pharynx. That these sensitive areas are so situated, is proven by their irritation by means of a probe. All the symptoms of hay fever can be produced, and, by their destruction by means of acid and cauteries, hay fever is cured. Also, by anesthetizing these areas by means of the muriate of cocaine, all the symptoms of "hay fever" are relieved.

Dr. Wm. Porter, of St. Louis, thought that nasal stenosis, when well treated, gave excellent results.

Dr. Chatham said it did not matter how much stenosis was present, we must have the irritation of the sensitive area before we can have hay fever.

